# Antimicrobial Resistance Patterns and Determinants of *Helicobacter pylori* Culture Success: A Prospective Study

**DOI:** 10.3390/antibiotics14121256

**Published:** 2025-12-12

**Authors:** Jee Young Sohn, Chang Seok Bang, A In Choi, Jeong-Gyu Choi, Eun Jeong Gong

**Affiliations:** 1 Department of Internal Medicine, Hallym University College of Medicine, Chuncheon 24253, Republic of Korea; jsohn92@hallym.or.kr (J.Y.S.); csbang@hallym.ac.kr (C.S.B.); 2 Institute of New Frontier Research, Hallym University College of Medicine, Chuncheon 24253, Republic of Korea; choiain@hallym.ac.kr (A.I.C.);

**Keywords:** culture, diagnosis, *Helicobacter pylori*

## Abstract

**Background/Objectives**: *Helicobacter pylori* infection remains a significant health concern, as increasing antimicrobial resistance compromises the efficacy of eradication. Understanding regional antimicrobial resistance profiles is crucial for optimizing eradication strategies. In this study, we aimed to evaluate the antimicrobial susceptibility patterns and identify the factors influencing *H. pylori* culture success. **Methods**: In this prospective study, 697 gastric tissue samples were collected from consecutive patients who underwent upper endoscopy between November 2023 and May 2025. Tissue samples obtained by forceps biopsy or recovered from rapid urease test kits were cultured for *H. pylori*. Antimicrobial susceptibility testing was performed using the agar dilution method; factors associated with successful culture were analyzed using logistic regression. **Results**: Among 488 patients with *H. pylori* infection, culture and antimicrobial susceptibility testing were successful in 387 (79.3%). The overall antimicrobial resistance rates were 17.8%, 27.1%, 29.5%, 0.3%, and 32.8% for amoxicillin, clarithromycin, metronidazole, tetracycline, and levofloxacin, respectively. Notably, 27.6% (107/387) of the isolates were resistant to two or more antibiotics. Using multivariate analysis, the use of fresh biopsy tissue (odds ratio [OR]: 1.646, 95% confidence interval [CI]: 1.046–2.591, *p* = 0.031), transport interval (OR: 0.911, 95% CI: 0.853–0.973, *p* = 0.005), and presence of prior eradication therapy (OR: 0.318, 95% CI: 0.156–0.648, *p* = 0.002) were identified as significant predictors of culture success. **Conclusions**: The high rate of clarithromycin resistance underscores the need for susceptibility-guided eradication strategies in this region. Optimizing sample handling, particularly by minimizing transport time and using fresh biopsy tissue, may improve culture yields.

## 1. Introduction

*Helicobacter pylori* is a Gram-negative bacterium that colonizes the human stomach, and infects approximately half of the global population [1]. *H. pylori* infection is associated with chronic gastritis, peptic ulcer disease, gastric mucosa-associated lymphoid tissue lymphoma, and non-cardia gastric adenocarcinoma [2]. Recent projections have indicated that 76% of all gastric cancers worldwide are attributable to *H. pylori* infection, with an estimated 15.6 million cases expected among individuals born between 2008 and 2017 [3]. This represents the largest preventable cancer burden caused by any infectious agent globally. As a treatable bacterial infection, *H. pylori* is a potentially modifiable risk factor for gastric cancer prevention [4,5]. However, its clinical management has become increasingly challenging owing to rapidly escalating antimicrobial resistance, which compromises the efficacy of the standard eradication regimens [6,7].

Global surveillance data have revealed alarming resistance patterns that have fundamentally shifted treatment paradigms worldwide. A comprehensive systematic review of 178 studies from 65 countries reported that the primary and secondary resistance rates to clarithromycin, metronidazole, and levofloxacin were ≥15% in all WHO regions, with increasing resistance observed in most regions [8]. The study also demonstrated a substantial increase in resistance to clarithromycin, metronidazole, and levofloxacin between 2013 and 2023. Although resistance prevalence varies across regions, the emergence of multidrug resistance contributes to up to 30% of first-line therapy failures, further complicating therapeutic efforts [9].

These evolving resistance patterns have prompted major revisions to the international treatment guidelines. The Maastricht VI/Florence consensus and recent American College of Gastroenterology guidelines now discourage empirical clarithromycin-containing regimens in regions where resistance exceeds 15% [10,11]. These guidelines emphasize susceptibility-guided therapy when feasible, particularly in patients with previous eradication failures, wherein resistance rates are substantially higher. When susceptibility testing is unavailable, treatment selection should be based on local antimicrobial resistance data. Therefore, understanding regional resistance profiles is crucial for optimizing empirical therapies and establishing appropriate treatment guidelines.

Bacterial culture is the most specific diagnostic method for *H. pylori* infection and remains essential for antimicrobial susceptibility testing for guiding therapy [12]. However, implementing culture-based approaches faces significant methodological obstacles that limit their widespread clinical application. Culture success rates show considerable variability; one study reported a success rate as low as 13.3%, whereas another found that 20% of attempts to determine antimicrobial resistance by culture were unsuccessful [13,14]. This variability presents substantial barriers to implementing tailored therapy and conducting reliable surveillance programs essential for monitoring resistance trends. Multiple factors influence culture success, including specimen handling, transport conditions, laboratory methodology, and patient-specific variables [13,15]. However, the relative impact of these factors and their optimal implementation remains poorly understood, requiring further systematic evaluation to inform evidence-based laboratory protocols.

Given the high global and regional antimicrobial resistance rates and clinical need for reliable susceptibility testing, there is an urgent need for identifying and optimizing factors that influence *H. pylori* culture success. Understanding these determinants is crucial for improving diagnostic accuracy and supporting the broader implementation of resistance-guided therapy in clinical practice. In this prospective study, we aimed to evaluate contemporary antimicrobial susceptibility patterns and systematically identify the factors affecting *H. pylori* culture success.

## 2. Results

### 2.1. Characteristics of the Study Population

During the study period, 697 gastric tissue samples were collected from patients who underwent endoscopy. Of these, 488 (70.0%) were diagnosed with *H. pylori* infection through at least one diagnostic modality. The baseline characteristics of the patients with *H. pylori* infection are presented in Table 1. The median patient age was 60 years (range, 21–87 years), with an approximately equal sex distribution. Chronic atrophic gastritis was the most prevalent endoscopic finding (65.5%), followed by peptic ulcer disease (15.4%), lymphofollicular gastritis (7.2%), gastric dysplasia (6.6%), and gastric cancer (5.3%). Open-type atrophic gastritis was present in 319 patients (65.4%), whereas 169 patients (34.6%) had closed-type atrophy. At the time of specimen collection, 135 patients (27.7%) were on acid-suppressive agents, such as proton pump inhibitors (PPIs; 13.7%) and potassium-competitive acid blockers (PCABs; 12.3%). A history of *H. pylori* eradication therapy was found in 36 patients (7.4%).

### 2.2. Culture and Antimicrobial Susceptibility Profiles

**Figure 1:**
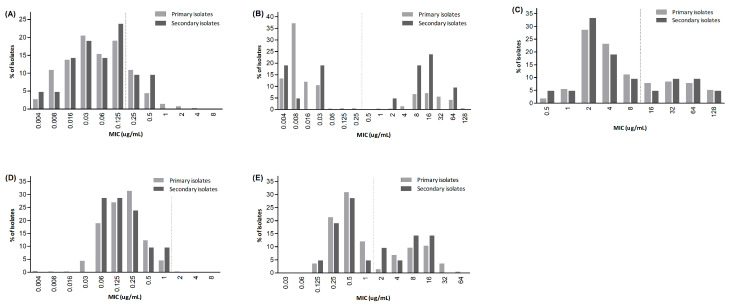
Distribution of minimum inhibitory concentration (MIC) of antibiotics: (**A**) amoxicillin, (**B**) clarithromycin, (**C**) metronidazole, (**D**) tetracycline, (**E**) levofloxacin. The dotted vertical line indicates the resistance breakpoint for each antibiotic.

*H. pylori* culture and antimicrobial susceptibility testing were successfully performed in 387 of the 488 *H. pylori*-positive specimens, yielding an overall culture success rate of 79.3%. Among the 366 primary isolates, the resistance rates to amoxicillin, clarithromycin, metronidazole, tetracycline, and levofloxacin were 17.8%, 25.4%, 29.5%, 0.3%, and 32.2%, respectively (Table 2). Among the secondary isolates (*n* = 21), the resistance rates were higher to clarithromycin (57.1%) and levofloxacin (42.9%), while the rates to other antibiotics were comparable (Figure 1).

**Figure 2:**
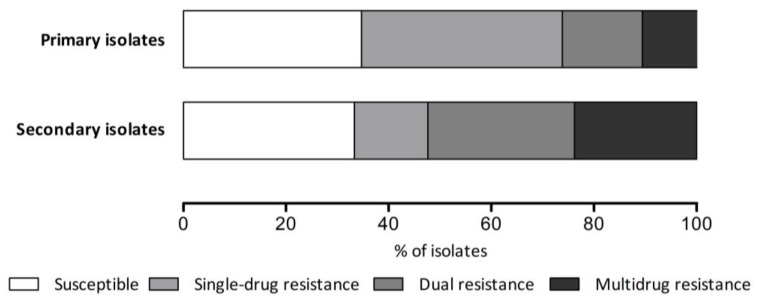
Antimicrobial resistance pattern according to eradication history.

Among all the isolates, 107 (27.6%) demonstrated resistance to two or more antibiotics. Dual resistance was observed in 63 isolates (16.3%), with the clarithromycin-levofloxacin combination (6.2%) being the most prevalent. Other common dual resistance patterns were metronidazole-levofloxacin (3.4%) and clarithromycin-metronidazole (2.6%). Among the multidrug-resistant isolates, the most frequent pattern was clarithromycin-metronidazole-levofloxacin (3.1%). Five isolates (1.3%) were resistant to four antibiotics. The secondary isolates showed a higher prevalence of both dual (28.6%) and multidrug resistance (23.8%) than did the primary isolates (Figure 2). The clarithromycin-levofloxacin dual resistance pattern was particularly common among the secondary isolates (19.0%), reflecting the use of these antibiotics in previous treatment attempts.

### 2.3. Factors Associated with Culture Success

The comparative characteristics of the successful and failed culture groups are detailed in Table 3. Among the 276 samples obtained by forceps biopsy, 227 (82.2%) were successfully cultured, compared with 160 of 212 (75.5%) samples recovered from the rapid urease test (RUT) kits (*p* = 0.072). The median transport time was significantly shorter for successful cultures than for failed ones (2.67 vs. 3.08 h, *p* = 0.001). A history of eradication therapy was strongly associated with culture failure. Among 452 treatment-naïve patients, culture was successful in 366 (81.0%), compared with 21 (58.3%) of the 36 patients receiving prior eradication therapy (*p* = 0.003). The use of acid-suppressive agents, presence of open-type atrophic gastritis, and intestinal metaplasia was not significantly associated with culture success.

Table 4 presents the factors associated with *H. pylori* culture success identified by logistic regression analysis of factors. Using univariate analysis, the tissue source, transport interval, and a history of eradication therapy were associated with culture success. Using multivariate analysis, all three variables remained independently associated with culture success. The use of fresh biopsy tissue was significantly associated with successful culture compared with the use of residual tissue from RUT kits (odds ratio [OR]: 1.646, 95% confidence interval [CI]: 1.046–2.591, *p* = 0.031). Each additional hour of transport time decreased the odds of successful culture by approximately 9% (OR: 0.911, 95% CI: 0.853–0.973, *p* = 0.005). A history of eradication therapy was the strongest negative predictor (OR: 0.318, 95% CI: 0.156–0.648, *p* = 0.002).

## 3. Discussion

In this prospective study, we evaluated the antimicrobial susceptibility patterns of *H. pylori* and identified the factors affecting its culture success. Among 488 patients with *H. pylori* infection confirmed by at least one diagnostic modality, culture and antimicrobial susceptibility testing were successful in 387 (79.3%). The success rate was significantly associated with shorter transport time, use of fresh biopsy tissue, and absence of prior eradication therapy. The identification of modifiable factors affecting culture success and documentation of high antibiotic resistance rates have implications for clinical practice.

Our findings demonstrated the successful implementation of culture-based susceptibility testing in a regional center, achieving a culture success rate of 79.3%. This finding aligns with that of a systematic review of 41 studies, which reported an average success rate of 80.1% [14]. Our rate is particularly noteworthy given that we included all consecutive samples rather than selected cases and used two different tissue sources, including specimens recovered from RUT kits. This high success rate validates our laboratory protocols and confirms that culture-based susceptibility testing is feasible in regional centers with appropriate technical expertise and infrastructure.

The successful isolation of *H. pylori* from gastric biopsy samples presents significant challenges, primarily owing to factors related to transport conditions, including duration, temperature fluctuations, and exposure to ambient air [16]. In this study, we focused on practical aspects of sample collection and transport that could be optimized in clinical practice to improve culture yields and facilitate the adaptation of susceptibility-guided therapy. We identified three independent factors associated with *H. pylori* culture success: shorter transport intervals, use of fresh biopsy tissue, and absence of prior eradication therapy.

Although no definitive cut-off time was established, culture success declined with longer transport intervals. Each hour of delay reduced the odds of successful culture by approximately 9%, reflecting the sensitivity of *H. pylori* to environmental conditions. In this study, the median transport time for successful culture was 2.67 h, aligning with that in previous reports that recommend processing within 4 h [17]. A large Chinese study including 66452 patients demonstrated a significantly lower culture success rate in samples processed within 48 h (26.3%) than in those processed within 24 h (33.7%) [18]. Similarly, a Canadian study found significantly improved culture success rates with short transport intervals, particularly less than 1 h [15]. Our findings also demonstrated a time-dependent decline: 80.8% (336/416) for specimens processed within 4 h, 74.2% (49/66) for those processed between 4 and 24 h, and 33.3% (2/6) for those exceeding 24 h, indicating that timely processing within 4 h significantly enhanced culture yield. These findings highlight the need for coordinated workflows between endoscopy units and microbiology laboratories, particularly in regional settings, where centralized processing may involve longer transport distances [19].

Fresh biopsy tissue yielded significantly higher culture success rates (82.2%) than did those recovered from RUT kits (75.5%). This finding has practical implications for clinical practice. Obtaining dedicated tissue for culture requires at least one additional biopsy beyond that required for RUT. Although endoscopic forceps biopsy is generally safe, the potential risks include bleeding and perforation [20,21]. Therefore, the decision to obtain additional biopsies should be individualized, particularly in patients at increased bleeding risk, including those with thrombocytopenia, coagulopathy, or those receiving anticoagulants or antiplatelet therapy. Although using RUT-recovered tissue eliminates the need for additional biopsies, our results suggest that dedicated fresh tissue samples should be obtained when culture-based testing is essential. Nevertheless, the 75.5% success rate with RUT-recovered tissue supports its utility as a viable alternative when additional biopsies are not feasible.

In our study, culture success rates were significantly lower in patients receiving prior eradication therapy (58.3%, 21/36) than in treatment-naïve patients (81.0%, 366/452). A history of eradication therapy also remained a strong negative predictor using multivariate analysis. A systematic review by Francesco et al., which analyzed 41 studies encompassing 7889 infected patients, reported culture success rates of 78.1% before first-line therapy, 77.5% before second-line therapy, and higher rates of 86.3% and 86.6% before third-line therapy and after multiple eradication failures, respectively [14]. Although the difference was not statistically significant, this review demonstrated a 0.6% decline in culture success rate after the first treatment failure, consistent with the directional trend observed in our study, but with a more pronounced reduction from 81.0% to 58.3%. Several factors may explain the reduced culture yield after prior eradication therapy. Prior antibiotic exposure can lower bacterial load; select for slower-growing, more fastidious strains; or induce viable but non-culturable coccoid forms, thereby reducing recovery rates [22]. This finding has important clinical implications, as patients with treatment failure are precisely those who would most benefit from susceptibility-guided therapy. In such cases, enhanced culture methods—such as obtaining copious mucosal samples via sweeping or enrichment techniques—may improve isolation success [23].

Clarithromycin-based *H. pylori* eradication therapies were introduced in the 1990s; global resistance rates to clarithromycin remained at or below 10% until the early 2000s [24]. However, resistance to clarithromycin has substantially increased, now exceeding the 15% threshold in all WHO regions, where the international guidelines discourage empirical clarithromycin-based therapy [10,11]. A landmark nationwide Korean study comprising 590 patients across 15 centers revealed a similar trend: clarithromycin resistance rates increased from 8.5% (2005–2010) to 16.0% (2011–2012), then to 17.8% (2017–2018), and reached 28.6% in some recent reports (2017–2019) [25]. Consistent with these findings, our study observed a clarithromycin resistance rate of 27.1%, substantially exceeding the 15% threshold beyond which empirical use is discouraged.

The current Korean guidelines recommend clarithromycin-containing triple therapy (PPI, amoxicillin, and clarithromycin—PAC regimen) as the first-line treatment for *H. pylori* eradication [26]. However, our findings suggest that this approach should be re-evaluated. With clarithromycin resistance exceeding 15% both in our study population and nationwide, empirical use of clarithromycin-based regimens without antimicrobial susceptibility testing is possibly suboptimal and may contribute to resistance development [25]. The markedly higher resistance rate of 57.1% observed in secondary isolates further supports the need for susceptibility testing before initiating rescue therapy [10]. Given that such testing is not universally accessible in routine clinical practice, revising the national guidelines to support non-clarithromycin-based regimens as empirical first-line therapy is warranted. Additionally, levofloxacin resistance rate exceeding 30% is concerning, as it compromises the efficacy of levofloxacin-based regimen [27]. The high prevalence of clarithromycin-levofloxacin dual resistance in this study limits both first-line and rescue therapy options and underscores the importance of susceptibility testing before using these antibiotics.

Consistent with global epidemiological trends, metronidazole resistance rates in Korea have progressively increased, exceeding 30% in recent nationwide and regional surveillance reports [25,28]. Unlike clarithromycin, where resistance strongly predicts treatment failure, the correlation between in vitro metronidazole resistance and clinical outcomes remains complex and often discordant. Evidence suggests that higher doses, prolonged treatment duration, or bismuth supplementation may help overcome metronidazole resistance [29,30,31,32]. Several studies have shown comparable results in *H. pylori* eradication rates in high metronidazole resistance settings when one or more of these strategies were employed: high-dose metronidazole or combination therapy with bismuth and other antibiotics, such as amoxicillin and tetracycline [33,34,35]. These findings suggest that metronidazole-based regimens remain feasible first-line treatment options in Korea when appropriately optimized for dose and duration, despite the increasing resistance rates.

This study has some limitations. First, as a single-center study, although our findings might not be generalizable to all regions, they are possibly to reflect patterns in similar provincial settings. Antimicrobial resistance patterns are well-known to show regional variation. These differences are found even within a single country, leading to different eradication success rates according to region [25,36]. Regional variations are influenced by multiple factors, including antibiotic consumption pattern, comorbidities, and local healthcare accessibility [37,38,39]. Expanding surveillance to multiple regional centers and investigating variables associated with local antimicrobial resistance in future studies would better elucidate geographic variations in resistance patterns. Second, since this study was conducted at a university hospital, a considerable proportion of patients were referred from primary care clinics or presented with multiple comorbidities, increasing the likelihood of prior antibiotic exposure. However, objective data on individual antibiotic usage were not analyzed. Future studies integrating prescription history or data on antibiotic consumption would further elucidate the epidemiology of local antimicrobial resistance. Third, the relatively small number of secondary isolates might limit the precision of resistance estimates in this subgroup. Lastly, we did not evaluate clinical outcomes based on susceptibility-guided therapy, which would strengthen the clinical relevance of our findings. As in vitro resistance does not always predict clinical treatment failure, real-world eradication rates might differ from those predicted solely by susceptibility testing. Although the current study provided essential baseline data on antimicrobial resistance patterns, prospective investigations evaluating eradication outcomes of susceptibility-guided therapy would further enhance the therapeutic application of our findings.

Despite these limitations, this study has several strengths. In South Korea, the national surveillance data have demonstrated increasing resistance to clarithromycin and levofloxacin over the past two decades; however, most studies have focused on metropolitan areas [25,28,40]. Regional data, particularly from less urbanized areas, remain limited despite potential differences in antimicrobial prescription patterns and treatment practices [36,41]. Gangwon Province, located in northeastern South Korea, represents a unique epidemiological setting with a predominantly rural population and limited data on *H. pylori* antimicrobial resistance patterns. The successful establishment of *H. pylori* cultures in our regional center demonstrates that susceptibility testing is feasible outside major metropolitan areas and is essential for addressing regional variations in resistance patterns. Moreover, the identified factors affecting culture success provide actionable targets for quality improvement initiatives. In this study, we focused on modifiable operational factors rather than unmodifiable characteristics. Although stratified analysis by demographic factors might provide additional insights, the primary barriers to culture success—transport time and tissue handling—represent practical targets that could be optimized across diverse patient populations.

In conclusion, we demonstrated that a regional center can successfully perform *H. pylori* culture and antimicrobial susceptibility testing, achieving a culture success rate of 79.3%. The key modifiable factors for optimizing culture success include minimizing transport time using fresh biopsy tissue when culture is essential and recognizing challenges posed by prior eradication therapy. The high rates of clarithromycin resistance along with substantial multidrug resistance underscore the need for susceptibility-guided therapy in our region. As antimicrobial resistance continues to evolve, region-specific treatment strategies and broader access to susceptibility testing beyond major centers are essential for maintaining effective *H. pylori* treatment strategies.

## 4. Materials and Methods

### 4.1. Study Population

In this prospective observational study, adult patients undergoing upper endoscopy for screening purposes or evaluation of gastrointestinal symptoms were recruited between November 2023 and May 2025. Patients were included if endoscopic findings indicated *H. pylori*-associated pathology, such as chronic atrophic gastritis, peptic ulcer disease, lymphofollicular gastritis, gastric dysplasia, or gastric cancer. The exclusion criteria were a history of gastric surgery, contraindications to biopsy, or inability to provide informed consent. Recent or current use of acid-suppressive agents (PPIs, PCABs, and H_2_-receptor antagonists), as well as a history of *H. pylori* eradication therapy, did not preclude participation. Demographic information, medical histories, and endoscopic findings were also documented. A history of *H. pylori* eradication therapy was verified through medical records and patient interviews, and isolates were classified as primary (no prior treatment) or secondary (previous treatment failure). The study protocol was approved by the Institutional Review Board of Hallym University Chuncheon Sacred Hospital (No. 2023-10-003); written informed consent was obtained from all participants. The study was conducted in accordance with the principles of the Declaration of Helsinki.

### 4.2. Endoscopic Procedure and Sample Collection

Upper endoscopy and sample collection were performed by two experienced gastroenterologists. Endoscopic findings were documented and classified according to the Kyoto classification [42]. Atrophic gastritis was classified as closed or open-type based on the Kimura-Takemoto classification [43]. The presence and extent of intestinal metaplasia were also documented. Gastric tissue samples for *H. pylori* culture were obtained from two sources: (i) forceps biopsy from the greater curvature of the corpus, antrum, or both, and (ii) tissue specimens recovered from RUT kits (Helicosign Dry; GenBody Inc., Cheonan, Republic of Korea) after completing the colorimetric reaction. Tissue specimens were placed in sterile tubes containing normal saline and stored at 4 °C until transport to the laboratory. Transport time was defined as the interval from specimen collection to laboratory receipt and was recorded for each sample.

### 4.3. H. pylori Diagnosis

*H. pylori* infection status was determined using multiple diagnostic methods to maximize sensitivity. The patients were classified as *H. pylori*-positive if any of the following tests yielded positive results: RUT, ^13^C-urea breath test, or bacterial culture. The patients were considered *H. pylori*-negative only if all tests showed negative results. A positive RUT result was defined as a change in color from yellow to red within 60 min at room temperature, whereas the result was considered negative if the color remained yellow after 60 min.

### 4.4. Bacterial Culture and Antimicrobial Susceptibility Testing

Regarding *H. pylori* isolation, each biopsy specimen was gently streaked onto selective Brucella agar (Difco; Becton Dickinson and Company, Sparks, MD, USA) plates supplemented with 7% defibrinated sheep blood (MBcell, Seoul, Republic of Korea) or heat-inactivated horse serum (Gibco;Thermo Fisher Scientific, Waltham, MA, USA), along with antibiotics (amphotericin B 5 μg/mL, vancomycin 10 μg/mL, trimethoprim 5 μg/mL, and polymyxin 2.5 IU/mL). All plates were incubated at 37 °C in a microaerophilic atmosphere (10% CO_2_) for 5–7 days. Suspected colonies were identified as *H. pylori* based on characteristic colony morphology and positive biochemical tests (urease, catalase, and oxidase). Additionally, a polymerase chain reaction (PCR) targeting the *glmM* gene was performed on each isolate using primers (forward sequence: 5′ AAG CTT TTA GGG GTG TTA GGG GTT 3′; reverse sequence: 5′ AAG CTT ACT TTC TAA CAC TAA CGC 3′) after DNA extraction with the AccuPower^®^ PCR pre-mix kit (Bioneer, Daejeon, Republic of Korea), as previously described [17]. Confirmed *H. pylori* isolates were subcultured and stored at −80 °C in tryptic soy broth containing 15% glycerol for further analysis.

Antimicrobial susceptibility testing of *H. pylori* isolates was performed using the agar dilution method on Mueller–Hinton agar (Difco) supplemented with 5% defibrinated sheep blood (MBcell) and two-fold serial dilutions of each antibiotic. The following antibiotics were tested: amoxicillin, clarithromycin, metronidazole, tetracycline, and levofloxacin. Bacterial suspensions were prepared in phosphate-buffered saline to a turbidity equivalent to approximately 2.0 McFarland standard. A 5 μL aliquot of this bacterial suspension was inoculated onto each antibiotic-containing agar plate. The inoculated plates were incubated at 37 °C under microaerophilic conditions and examined after 72 h. The minimum inhibitory concentration (MIC) was defined as the lowest concentration that completely inhibited visible bacterial growth. *H. pylori* ATCC 43504 served as the quality control strain.

Resistance breakpoints were defined according to the European Committee on Antimicrobial Susceptibility Testing guideline: >0.125 μg/mL, >0.25 μg/mL, >8 μg/mL, >1 μg/mL, and >1 μg/mL for amoxicillin, clarithromycin, metronidazole, tetracycline, and levofloxacin, respectively [44]. Isolates with MICs above these thresholds were classified as resistant to the respective antibiotics. Isolates were further classified as having single drug resistance (resistant to one antibiotic), dual resistance (resistant to two antibiotics), or multidrug resistance (resistant to three or more antibiotics).

### 4.5. Statistical Analysis

Descriptive statistics were used to summarize the data. Categorical variables are presented as counts and percentages, while continuous variables are presented as medians with ranges. Regarding comparison, Student’s *t*-test, Mann–Whitney *U* test, chi-square test, or Fisher’s exact test was used as appropriate. Factors associated with culture success were analyzed using logistic regression. Variables with *p* < 0.1 in univariate analysis were included in the multivariate model, which was performed using backward stepwise elimination. ORs with 95% CIs were calculated. All analyses were performed using SPSS version 29.0 (IBM Corp., Armonk, NY, USA); statistical significance was set at *p* < 0.05.

## Figures and Tables

**Table 1:** Demographic and clinical characteristics of the study population.

Variables	*n* = 488
Age (years)	60 (21–87)
Male sex	251 (51.4%)
Endoscopic diagnosis	
Chronic atrophic gastritis	320 (65.6%)
Peptic ulcer diseases	75 (15.4%)
Lymphofollicular gastritis	35 (7.2%)
Gastric cancer	26 (5.3%)
Gastric dysplasia	32 (6.6%)
Acid suppressive agents use	
No	353 (72.3%)
Proton pump inhibitors	67 (13.7%)
Potassium-competitive acid blockers	60 (12.3%)
Histamine-2 receptor antagonists	8 (1.6%)
Atrophic gastritis	
Closed type	169 (34.6%)
Open type	319 (65.4%)
Intestinal metaplasia	203 (41.6%)
Previous eradication therapy	36 (7.4%)

Data represent median (range) or number (percent).

**Table 2:** Antimicrobial resistance profiles stratified by eradication history.

	Primary Isolates (*n* = 366)	Secondary Isolates (*n* = 21)	Total (*n* = 387)
Amoxicillin	65 (17.8%)	4 (19.0%)	69 (17.8%)
Clarithromycin	93 (25.4%)	12 (57.1%)	105 (27.1%)
Metronidazole	108 (29.5%)	6 (28.6%)	114 (29.5%)
Tetracycline	1 (0.3%)	0	1 (0.3%)
Levofloxacin	118 (32.2%)	9 (42.9%)	127 (32.8%)
**Dual resistance**	57 (15.6%)	6 (28.6%)	63 (16.3%)
A, C	8 (2.2%)	0	8 (2.1%)
A, M	7 (1.9%)	1 (4.8%)	8 (2.1%)
C, M	9 (2.5%)	1 (4.8%)	10 (2.6%)
C, L	20 (5.5%)	4 (19.0%)	24 (6.2%)
M, L	13 (3.6%)	0	13 (3.4%)
**Multidrug resistance**	39 (10.7%)	5 (23.8%)	44 (11.4%)
A, C, M	5 (1.4%)	0	5 (1.3%)
A, C, L	9 (2.5%)	2 (9.5%)	11 (2.8%)
A, M, L	10 (2.7%)	0	10 (2.6%)
C, M, L	10 (2.7%)	2 (9.5%)	12 (3.1%)
A, C, M, L	4 (1.1%)	1 (4.8%)	5 (1.3%)
A, M, T, L	1 (0.3%)	0	1 (0.3%)

A, amoxicillin; C, clarithromycin; L, levofloxacin; M, metronidazole; T, tetracycline.

**Table 3:** Patient and clinical characteristics by culture outcome.

	Fail (*n* = 101)	Success (*n* = 387)	*p* Value
Age (years)	62 (21–85)	59 (22–87)	0.415
Endoscopic diagnosis			0.185
Chronic atrophic gastritis	65 (20.3%)	255 (79.7%)	
Peptic ulcer diseases	14 (18.7%)	61 (81.3%)	
Lymphofollicular gastritis	4 (11.4%)	31 (88.6%)	
Gastric cancer	7 (26.9%)	19 (73.1%)	
Gastric dysplasia	11 (34.4%)	21 (65.6%)	
Characteristics of sample			
Location in the stomach			0.525
Antrum	4 (26.7%)	11 (73.3%)	
Corpus	97 (20.5%)	376 (79.5%)	
Source of tissue			0.072
Leftover tissue after RUT	52 (24.5%)	160 (75.5%)	
Biopsy	49 (17.8%)	227 (82.2%)	
Transport interval (h)	3.08 (0.33–52.00)	2.67 (0.57–27.00)	0.011
Acid suppressive agents use			0.599
No	73 (20.7%)	280 (79.3%)	
Proton pump inhibitors	12 (17.9%)	55 (82.1%)	
Potassium-competitive acid blockers	13 (21.7%)	47 (78.3%)	
Histamine-2 receptor antagonists	3 (37.5%)	5 (62.5%)	
Atrophic gastritis			0.725
Closed type	33 (19.5%)	136 (80.5%)	
Open type	68 (21.3%)	251 (78.7%)	
Intestinal metaplasia	46 (22.7%)	157 (77.3%)	0.367
Previous eradication therapy	15 (41.7%)	21 (58.3%)	0.003

RUT, rapid urease test.

**Table 4:** Factors associated with successful culture.

	Univariate Analysis		Multivariate Analysis	
	Odds Ratio (95% CI)	*p* Value	Odds Ratio (95% CI)	*p* Value
Biopsy tissue (vs. residual tissue after RUT)	1.506 (0.970–2.337)	0.072	1.646 (1.046–2.591)	0.031
Transport interval (h)	0.920 (0.862–0.981)	0.011	0.911 (0.853–0.973)	0.005
Acid suppressive agents use (vs. non-user)	0.966 (0.611–1.625)	0.988		
Open type atrophic gastritis	0.896 (0.562–1.426)	0.725		
Presence of intestinal metaplasia	0.816 (0.525–1.269)	0.367		
Previous eradication history	0.329 (0.163–0.664)	0.003	0.318 (0.156–0.648)	0.002

CI, confidence interval; RUT, rapid urease test.

## Data Availability

The original data presented in the study are available in the article. Further inquiries can be directed to the corresponding author upon reasonable request.

## Abbreviations

The following abbreviations are used in this manuscript:

MIC	Minimum inhibitory concentration
PCAB	Potassium-competitive acid blockers
PPI	Proton pump inhibitors
RUT	Rapid urease test
OR	Odds ratio
CI	Confidence interval
